# DNA Polymerase-Parental DNA Interaction Is Essential for Helicase-Polymerase Coupling during Bacteriophage T7 DNA Replication

**DOI:** 10.3390/ijms23031342

**Published:** 2022-01-25

**Authors:** Chen-Yu Lo, Yang Gao

**Affiliations:** Department of BioSciences, Rice University, Houston, TX 77005, USA; cl111@rice.edu

**Keywords:** DNA replication, helicase, polymerase, bacteriophage T7

## Abstract

DNA helicase and polymerase work cooperatively at the replication fork to perform leading-strand DNA synthesis. It was believed that the helicase migrates to the forefront of the replication fork where it unwinds the duplex to provide templates for DNA polymerases. However, the molecular basis of the helicase-polymerase coupling is not fully understood. The recently elucidated T7 replisome structure suggests that the helicase and polymerase sandwich parental DNA and each enzyme pulls a daughter strand in opposite directions. Interestingly, the T7 polymerase, but not the helicase, carries the parental DNA with a positively charged cleft and stacks at the fork opening using a β-hairpin loop. Here, we created and characterized T7 polymerases each with a perturbed β-hairpin loop and positively charged cleft. Mutations on both structural elements significantly reduced the strand-displacement synthesis by T7 polymerase but had only a minor effect on DNA synthesis performed against a linear DNA substrate. Moreover, the aforementioned mutations eliminated synergistic helicase-polymerase binding and unwinding at the DNA fork and processive fork progressions. Thus, our data suggested that T7 polymerase plays a dominant role in helicase-polymerase coupling and replisome progression.

## 1. Introduction

The precise and efficient replication of the DNA genome is fundamental to all living systems [1,2,3]. Double-stranded (ds) DNA is unzipped by the motor protein DNA helicase, producing two daughter strands that serve as templates for DNA synthesis. The fork structure formed by parental DNA and the two daughter strands is called a replication fork [4,5]. Because DNA synthesis is unidirectional following the 5′ to 3′ direction by DNA polymerases, only one strand of the parental dsDNA, the leading strand, can be synthesized continuously, while the lagging-strand polymerase extends the DNA as ~1 kb segments, called Okazaki fragments [1,2,3]. The single-stranded (ss) DNA intermediates during replication are protected by the ssDNA binding protein (SSB) [6]. However, since the DNA polymerase cannot start DNA synthesis de novo, it must rely on a primase to provide the primer with a free 3′-OH end to enable extension [1]. The polymerases, helicase, primase, SSB, and accessory proteins constitute an integral complex called the replisome [1,2,3]. This molecular machinery coordinates the operation of the helicase with DNA polymerases and other proteins to unwind parental DNA and synthesize both the leading and lagging strands. The helicase and leading-strand polymerase are core components in the multiple-protein complex. They perform dsDNA unwinding and leading-strand DNA synthesis cooperatively, thereby setting the pace for replisome progression [7,8,9,10,11]. Moreover, helicase-polymerase coupling is essential for the replisome to handle various challenges during replication. For example, uncoupling the helicase and leading-strand polymerase stalls replication and induces stress responses [12,13].

DNA helicases are chemo-mechanical motors that use the energy from the hydrolysis of triphosphate nucleotide (NTP) to power DNA unwinding [4,5,14]. Almost all replicative DNA helicases are hexameric and attributed to three superfamilies (SFs)—SF3, SF4, and SF6—which are involved in viral, bacterial, and eukaryotic and archaeal DNA replication, respectively [4,5]. SF3 and SF6 helicases are ATPases associated with diverse cellular activities (AAA+). These ATPases migrate in the 3′- to 5′-direction on the leading strand, whereas bacterial SF4 helicases belong to the RecA family and travel in the 5′- to 3′-direction on the lagging strand [5,14]. Such hexameric helicases usually have a C-terminus domain encoding the helicase and an N-terminal domain (NTD) for oligomerization, DNA binding, protein-protein interactions, and primer synthesis [4,14]. Helicases always place their N-terminal domain at the 5′-side of DNA and its C-terminal domain (ATPase region) at the 3′-side [4,14]. Even though the sequences and polarity of bacterial and eukaryotic helicases are different, structural, biochemical, and biophysical studies suggest that all hexameric helicases use a hand-over-hand sequential translocation mechanism [4,14]. Hexameric helicases form a ring- or lock-washer-like structure where ssDNA binds at the central channel of the ring, and the NTP molecules are located at the subunit interfaces [4,14]. Sequential NTP hydrolysis along the ring powers the translocation of the DNA binding motif or the entire subunit, along the ssDNA strand, end-to-end [15,16]. The translocated subunit can pull ssDNA and sterically exclude the complementary strand to drive DNA unwinding [14,15].

DNA polymerases catalyze template-dependent DNA synthesis with the help of metal ion cofactors [17,18]. Replicative polymerases often contain an exonuclease domain or subunit to proofread the newly synthesized DNA [17,19]. In addition, polymerases are associated with processivity factors that interact with primer-template dsDNA to ensure continuous DNA synthesis [20]. Based on sequences, replicative DNA polymerases in bacteria, archaea, eukaryotes, and bacteriophages are classified into C-family, B- or D-family, B-family, and A- or B-family polymerases, respectively [21]. Eukaryotic cells use different polymerases for leading- and lagging-strand DNA synthesis, while multiple copies of one type of polymerase are sufficient for bacterial and bacteriophage replication [1,2,3]. Although without sequence homologies, polymerase structures assume a right-handed architecture, with the active site in the palm domain. The primer-template dsDNA is stabilized by a thumb domain, and the nascent, incoming nucleotide-template base-pairs are enclosed by a finger domain [19]. An open-to-close conformational change of the finger domain has been observed after correct dNTP binding [22]. In addition, the translocation of the primer-template following DNA synthesis is also coupled to finger domain opening and closing [19,22,23].

The helicase and leading-strand polymerase work synergistically during DNA replication [1,2,3,7,8,9,10]. In bacterial and bacteriophage systems, the helicase and polymerase are on different strands at the replication fork between the two enzymes [24]. In a eukaryotic replisome, both polymerase and helicase are on the leading strand. The replication fork enters the NTD of the helicase, while the polymerase resides on the C-terminal side of the helicase [25,26]. Nevertheless, the actions of helicases and polymerases are coupled in all replisomes [7,11]. The helicase enhances polymerase processivity and activity, whereas the polymerase stimulates helicase unwinding [8]. Both the helicase and polymerase can translocate on ssDNA; however, the helicase alone can only unwind dsDNA inefficiently with frequent backtracking, while the polymerase alone exhibits low processivity and frequent exonuclease cleavage during strand-displacement synthesis [8,27,28]. Moreover, unwinding by an individual protein is passive and highly dependent on the base-pairing energy of parental DNA. When the helicase and polymerase are coupled at the replication fork, their unwinding is rapid, processive and will not be rate-limited by base-pair separation during fork progression [7,27].

**Figure 1:**
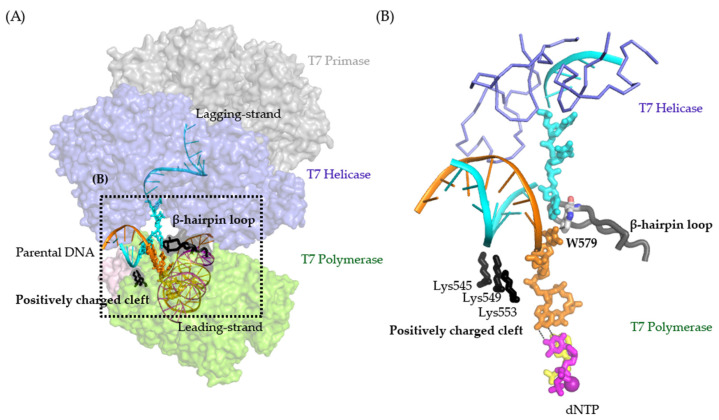
(**A**) Structure of T7 replisome on a DNA fork. The polymerase, helicase, and primase are colored green, blue, and grey, respectively. The positively charged cleft and β-hairpin loop of the T7 Polymerase are shown as black cartoons. The charged–charge interactions between the helicase and polymerase are indicated by pink and blue symbols. (**B**) Zoomed-in view of the DNA fork bound by the T7 polymerase and the helicase. The β-hairpin and the positively charged cleft (Lys545, Lys549, and Lys553) are depicted as a grey cartoon and black sticks.

The replisome from bacteriophage T7 is one of the simplest and has been a model system for investigating DNA replication [2]. The T7 replisome only requires the bifunctional gp4 helicase-primase, gp5 polymerase, host processivity factor trx, and gp2.5 SSB to function normally. Gp4 contains a primase on the NTD for primer synthesis and an SF4 family helicase on its C-terminus for DNA unwinding [29]. Gp5 is an A-family DNA polymerase and exhibits polymerase and exonuclease proofreading activity [30]. Trx, a host processivity factor, can bind to gp5 to form a gp5-trx holoenzyme complex and enhance the processivity of DNA synthesis [31,32]. During DNA replication, gp2.5 is the T7 SSB that interacts with gp4 and gp5 to coordinate their actions [33]. We recently captured the first structure of a replisome on a fork DNA substrate, using the T7 system [24]. In the structure, the T7 replisome formed a multiple-layer architecture with the leading-strand gp5 on the C-terminal helicase side of gp4 and the lagging-strand gp5 on the N-terminal primase side of the gp4. The leading-strand gp5 interacted the with flexible C-terminal tails of gp4. Meanwhile, the parental DNA sat between the helicase and leading-strand gp5, with the leading-strand ssDNA entering the gp5 active site and the lagging-strand ssDNA entering the helicase DNA binding channel. The two DNA strands ran in opposite directions and were ~90 degrees relative to the parental DNA. The structure suggested that the parental duplex was unzipped by cooperative pulling by the helicase and polymerase. However, the gp4 helicase within a replisome does not directly bind to the parental duplex or fork [24]. Instead, the leading-strand gp5 polymerase provides an intercalating β-hairpin loop with W579 to stack at the fork opening. Moreover, the gp5 polymerase encompasses a positively charged cleft with three lysine residues holding the parental duplex (Figure 1A,B). Previous studies suggested that the β-hairpin loop of gp5 possibly stabilizes the template during DNA synthesis and exonuclease proofreading [34]. However, the role of these two elements during coupled helicase-polymerase replication has not been determined. In this study, we created and characterized gp5 variants with a defective β-hairpin loop or positively charged cleft. Results from our biochemical and biophysical assays indicated that an intact β-hairpin loop promotes DNA binding and facilitates helicase-polymerase coupling during replication, whereas the positively charged cleft is essential for stabilizing the T7 replisome for processive DNA synthesis. Our results highlighted the unprecedented role of the polymerase in duplex unwinding during DNA synthesis.

## 2. Results

### 2.1. DNA Synthesis by Wild-Type (WT) and Mutant gp5-trx

**Figure 2:**
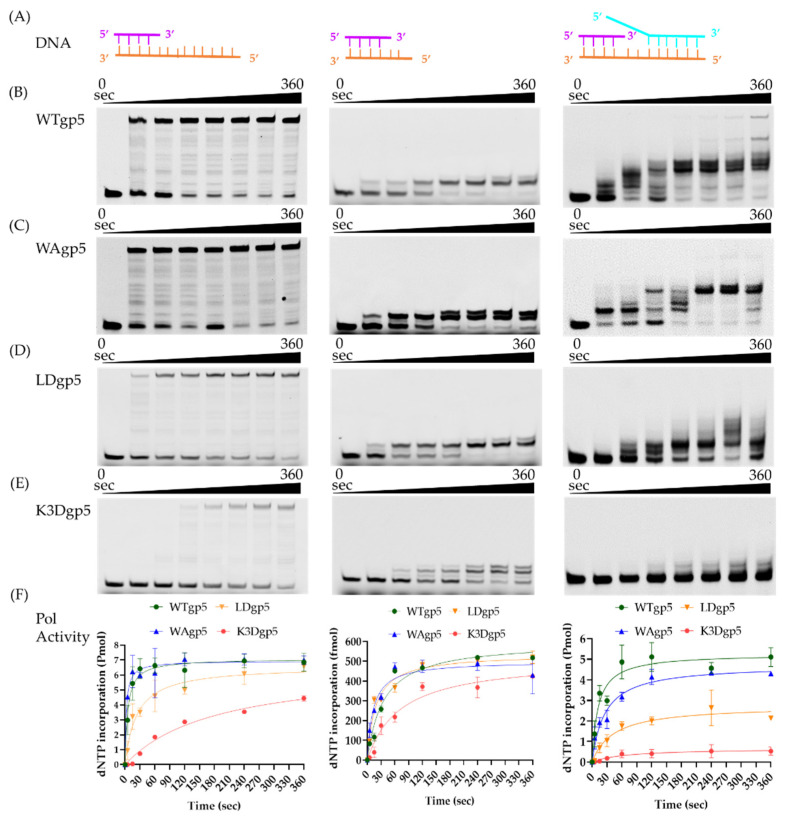
DNA polymerase activity of gp5 and its mutants. (**A**) Substrate design. (**B**–**E**) Gel results of DNA synthesis assays for WT (**B**), WA (**C**), LD (**D**), and K3D (**E**) gp5, respectively. (**F**) Quantification of DNA synthesis. DNA synthesis was estimated by measuring the amount of dNTP incorporation into nascent DNA over time as in the material and method section. The left, middle, and right panels correspond to results from linear, short linear, and fork DNA substrates, respectively.

To investigate the molecular basis of cooperative DNA unwinding and synthesis, we constructed T7 gp5 with positively charged cleft mutants K545A, K549A, and K553A (K3A) and K545D, K549D, and K553D (K3D). The K3A mutant was poorly expressed and had no polymerase activity, which was possibly due to improper folding. In addition, we created a loop mutation gp5 with W579Agp5 (WAgp5) and a loop deletion gp5 with the β-hairpin loop replaced by SGSG linker (LoopDel gp5 or LDgp5). We first tested the impacts of the positively charged cleft and β-hairpin loop on DNA polymerase activity using a linear DNA substrate consisting of a 26 bp duplex and 32 nucleotide (nt) overhang (Figure 2A). As shown in the left panel of Figure 2B–F, with WTgp5-trx, the full-length DNA synthesis product accumulated over time and reached a plateau at ~60 s. The WAgp5-trx did not significantly decrease DNA synthesis with the linear template. However, LD or K3D gp5-trx resulted in a moderate decrease (~1.5–2 fold) in gp5 DNA synthesis. To confirm that the reduced activity was due to attenuated interaction with downstream DNA, rather than protein folding or catalysis, we tested a DNA substrate with only two nucleotides overhanging (Figure 2A, middle). In the middle panel of Figure 2B–F, we found that the WT and mutant gp5-trx (WAgp5, LDgp5, and K3Dgp5) showed no significant difference in the incorporation of the two-nucleotide overhang on the short DNA template. Next, we compared the strand-displacement DNA synthesis of WT and mutant gp5-trx (WAgp5, LDgp5, and K3Dgp5) using a fork DNA template, consisting of 30 bp dsDNA and a 55 nt lagging strand overhang based on the same linear template (Figure 2A, right). Consistent with previous reports, the WTgp5-trx performed strand-displacement synthesis, but with reduced efficiency and lower processivity than when working with gp4 [31]. While WAgp5-trx activity was still comparable to that of WT, the LD or K3D gp5-trx DNA synthesis was significantly reduced (right panel of Figure 2B–F). Moreover, the final product length was shorter for LDgp5 and K3Dgp5 than WT. Therefore, these results suggested that both the positively charged cleft and the β-hairpin loop are required for downstream DNA unwinding but not for gp5 protein folding or catalysis, which confirmed the validity of our structural model.

### 2.2. DNA Binding by WT and Mutant gp5-trx

**Figure 3:**
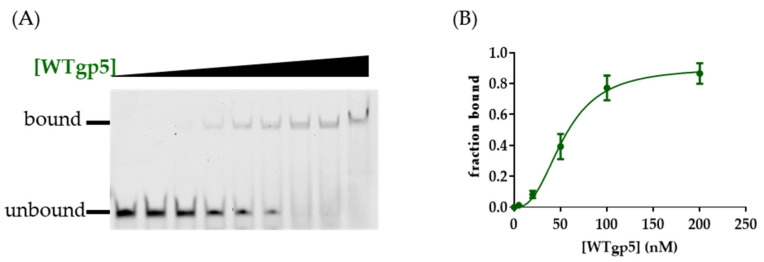
(**A**) Gel electrophoresis results of EMSA. (**B**) Quantification of the EMSA binding by fitting data to the Hill equation.

We next used gel mobility shift assay (EMSA) to measure the binding affinity of WT and mutant gp5 to various DNA substrates (Figure 3; Table 1; Figure S1). WTgp5-trx bound tightly to the linear primer-template with a K_d_ value of 53 nM, similar to what was reported [35]. Within the replisome structure, gp5-trx interacted with the 2 nt ssDNA between the primer end and fork: one for templating the incoming nucleotide and the other bound by gp5 (Figure 1B) [24]. The presence of a downstream fork and dsDNA with a 2 nt ssDNA gap on the leading strand enhanced gp5-trx binding (K_d_ of 36 nM) (Table 1). A shorter gap of 1 or 0 nt decreased the K_d_ to 63 and 110 nM, respectively (Table 1). We speculated that the decreased binding affinity with short-gapped DNA could be due to the energy required for duplex unwinding. To test whether the β-hairpin loop and positively charged cleft would affect the DNA binding affinity of gp5, we performed the same DNA binding assays with gp5 mutants. K3D gp5 showed ~2 to 4-fold weaker binding to both linear and fork DNA compared with WT (Table 1). WAgp5 showed similar binding affinities for the linear and fork DNA substrate compared to WTgp5 (Table 1). However, the DNA binding affinities of WAgp5 for gap 1 and 0 substrates were similar. LD gp5 bound linear DNA with a 2-fold lower affinity compared with WTgp5. The K_d_ value of LDgp5 remained at ~130 nM in the presence of a 0, 1, or 2 nt leading-strand ssDNA gap (Table 1).

### 2.3. Synergistic Hel-Pol Binding to Fork DNA

**Figure 4:**
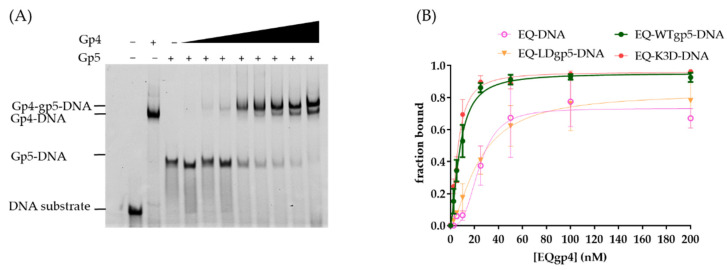
Cooperative gp4-gp5 binding to the fork template. (**A**) A representative gel of mobility shift assay for gp4-gp5 binding. (**B**). Quantification of the supershift gp4 binding to DNA or gp5-DNA. Legends suggested the type of gp4 and gp5 variants used in the binding assay.

Previous studies indicated that gp4 used its C-terminal tails to interact with the surface patches of gp5 at the replication fork [9,24]. To estimate whether the positively charged cleft and the β-hairpin loop also affected gp4 and gp5 assembling on a fork DNA, we used EMSA to measure the synergistic binding of gp5-trx and gp4. To simplify the binding condition, the gp4 active site mutant E343Q gp4 (EQgp4) was used because it has no hydrolysis but binds tightly to DNA [16,36]. EQgp4 bound to the fork DNA with a K_d_ of 24 nM in the presence of dTTP. When the DNA template was pre-bound to gp5-trx variants (60–100 nM), adding EQgp4 resulted in a supershifted band corresponding to the gp4-gp5-trx-DNA complex (Figure 4A). Furthermore, the presence of gp5-trx enhanced the binding affinity of EQgp4 (K_d_ 8 nM) and formed an EQgp4-gp5-trx-DNA complex. To confirm that the synergistic binding of gp4 and gp5-trx was due to their direct interaction, we used truncated C-terminal tails EQgp4 (EQgp4ΔC) in EMSA with gp5-trx. Deletion of the C-terminal tail had a minor effect on gp4 DNA binding (K_d_ value of 30 nM), which was similar to the K_d_ of full-length gp4 (Table 2; Figure S2). However, the binding was unchanged with or without gp5, suggesting that a direct gp4-gp5-trx interaction was essential for their cooperative binding to the replication fork. In addition, even with reduced DNA binding, K3D gp5-trx could also stimulate EQgp4 binding to the fork substrate (Figure 4B; Table 2; Figure S2). However, the LDgp5-trx failed to facilitate EQgp4 binding, which was analogous to the effect of deleting the C-tails of gp4 (Figure 4B; Table 2; Figure S2).

### 2.4. Cooperative gp4-gp5 Binding Induces Fork Unwinding

**Figure 5:**
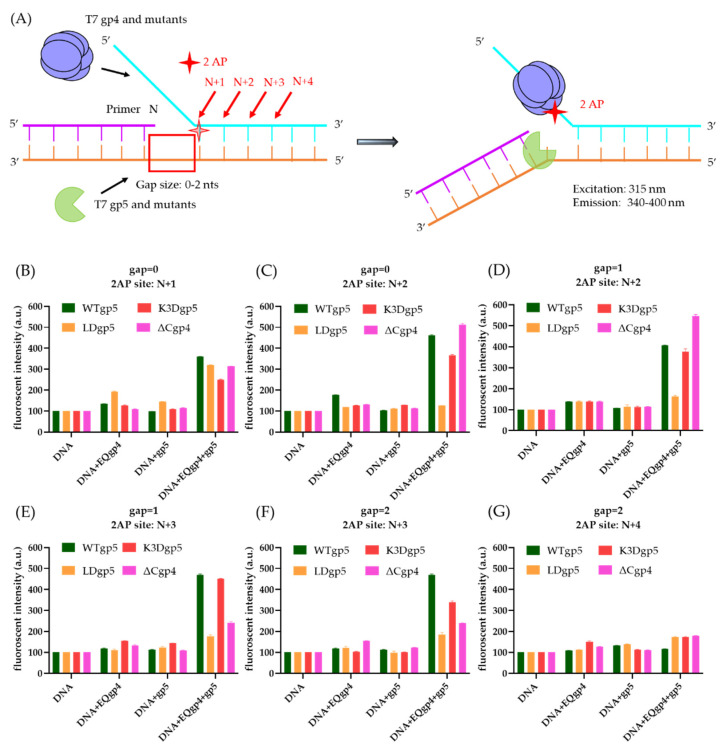
Cooperative separation of base pairs at the fork junction by gp4 and gp5-trx. (**A**) Design the replication fork with 2-AP labeled on the lagging strand. The primer-end (N) and subsequent base pairs on the duplex were N + 1 to N + 4. The 2-AP was shown as a red sticker on the duplex of the fork. (**B**–**G**) Integration of the fluorescent intensities of 2-AP modified substrates with or without gp5-trx and gp4. The gap size and the location of the 2-AP were indicated in each panel. Standard deviations were collected from the average of three replicates.

The biochemical data presented above indicated that the helicase and polymerase help each other to bind to a DNA fork. It was shown that the synergistic binding of gp4 and gp5 destabilized the fork and induced local fork unwinding [8]. Similar to the previous report, we used 2-aminopurine (2-AP) as a fluorescent probe to estimate the local base-pair unwinding. When 2-AP was base paired to thymidine (T), the DNA template had low fluorescence intensity, but the fluorescent signal rose when the base pair was melted. We designed a series of substrates with different gap sizes (0 to 2 nt) and 2-AP at various locations (N + 1 to N + 4, relative to the 3′-end of the primer) on the fork (Figure 5A). The 2-AP signal from the gp4 variants, gp5 variants, and the gp4-gp5 complexes in the reaction, respectively, were used to monitor cooperative base-pair unwinding. The gp4 or gp5-trx alone did not significantly change the fluorescence intensity levels with all substrates. In contrast, the gp4-gp5-trx complex caused elevated 2-AP fluorescence when the 2-AP was labeled at the N + 1, N + 2, and N + 3 positions, thereby suggesting a synergetic effect on duplex separation (Figure 5B–G). This signal was lowered to the background level when the 2-AP was labeled at the N + 4 position. The results were consistent with that of the previous report [8].

We next tested the local 2-AP unwinding with LDgp5-trx, K3Dgp5-trx, and EQgp4ΔC. When the 2-AP: T was at N + 1 position within the fork junction, the LDgp5-trx could still work with EQgp4 to unwind the first base pair. However, when the 2-AP: T was at N + 2 to N + 4, the EQgp4-LDgp5-trx complex failed to melt the base pair (Figure 5C–G). EQgp4ΔC also reduced cooperative melting but to a lesser extent. Observed fluorescent intensity of EQgp4ΔC-WTgp5 complex had a ~2-fold lower intensity at N + 3, indicating a decreased efficiency of base-pair unwinding compared with the EQgp4-WTgp5 complex (Figure 5E,F). Interestingly, the EQgp4-K3Dgp5-trx complex behaved similarly as the EQgp4-WTgp5-trx complex during duplex unwinding, with only 10–20% reduced fluorescent intensity compared to WTgp5 when the 2-AP: T was located at the N + 3 position.

### 2.5. Coupled DNA Synthesis by T7 Replisome

**Figure 6:**
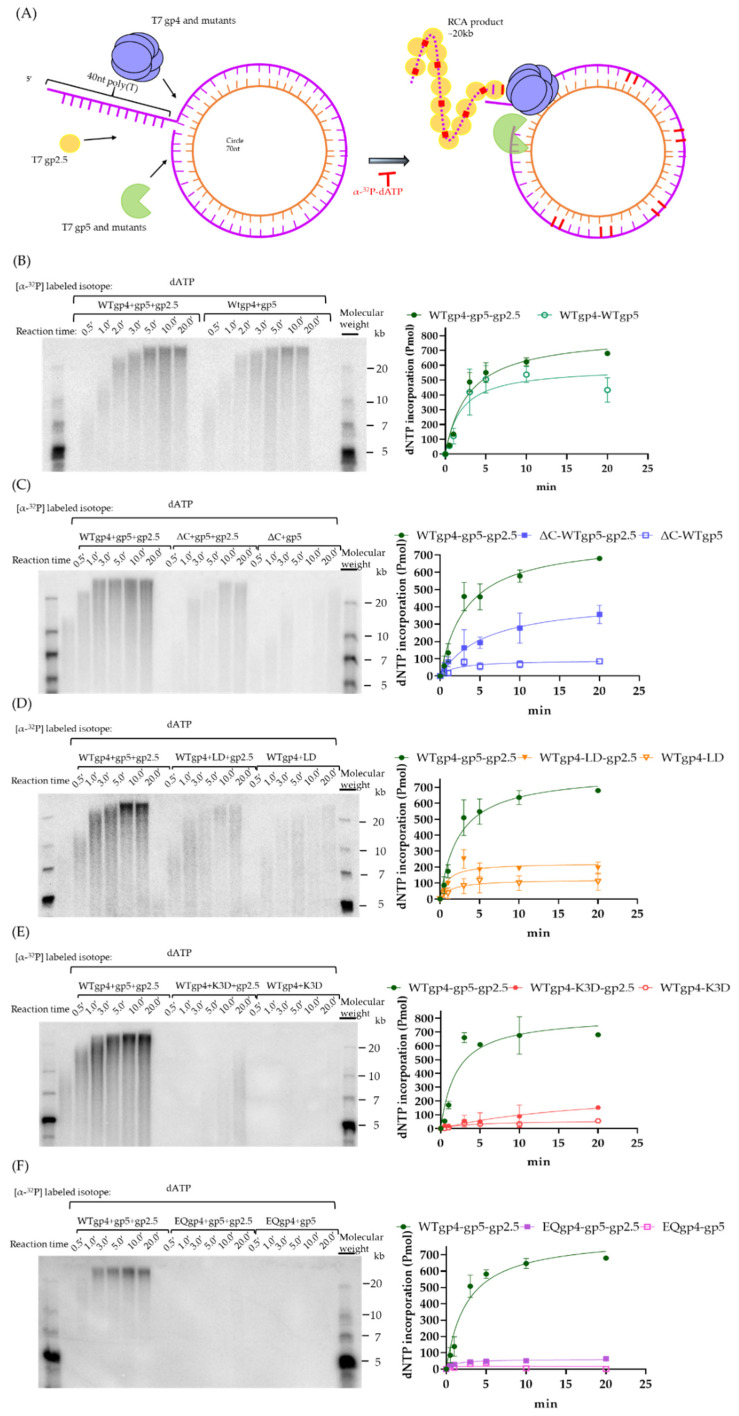
Rolling circle assay. (**A**) Structure of the minicircular template and the annealed primer. As illustrated in Materials and Methods, the duplex DNA was constituted by a 70 nt minicircular ssDNA and a 110 nt primer with 40 nt 5′ poly(T) for the loading of gp4. (**B**–**F**) Rolling circle amplification on the leading strand in the presence and absence of gp2.5 with WTgp4 and WTgp5 (**B**), ΔCgp4 and WTgp5 (**C**), WTgp4 and LDgp5 (**D**), WTgp4 and K3Dgp5 (**E**), and EQgp4 and WTgp5 (**F**). The gel results are on the left while the quantification is on the right. The WTgp4-WTgp5-trx-gp2.5 was used as controls in (**C**–**F)**. Color legends that reveal the gp4 and gp5 variants are on the right of the quantification graph.

Next, we investigated the effects of gp4 and gp5 variants on processive DNA replication. As shown in Figure 6A, we used a rolling circle assay based on a 70 nt minicircular template to estimate the efficiency of strand-displacement DNA synthesis driven by gp5-trx, gp4, and gp2.5 [37]. The α-^32^P-dATP was used to quantify the amount of dNTP incorporation over time. In this experiment, gp4, gp5-trx, and gp2.5 constituted the leading strand replisome and could support DNA synthesis with products up to a length of 20 kb (Figure 6B), but omitting gp2.5 reduced the replication product by 20%. However, the size of the RCA products remained at ~20 kb even when gp2.5 was omitted. In support of a previous study [9], our RCA result suggested that the truncation of the C-terminal tails of gp4 reduced the efficiency of strand-displacement DNA synthesis to ~50 and 12% in the presence and absence of gp2.5, respectively (Figure 6C). The complex of WTgp4-LDgp5-trx-gp2.5 showed a 2-fold (~200 pmol) lower efficiency during leading-strand DNA synthesis, and the DNA products were heterogeneous with a shorter size of 10–20 kb (Figure 6D). As shown in Figure 6E, with K3D gp5 the dNTP incorporation was reduced to only 14% (120 pmol) of the gp4-WTgp5-trx. Moreover, the size of the RCA products was smaller than 12 kb and heterogeneous. These results suggested the important role of the polymerase in coupled DNA synthesis. It has been recently suggested that helicase ATP hydrolysis is not required for bacterial replisome progression [38]. We used EQgp4 to assess whether the dTTP hydrolysis of gp4 was vital for efficient DNA synthesis. As shown in Figure 6F, operating with EQgp4 and WTgp5-trx, the T7 replisome can incorporate a mere ~55 pmol dNTP over time, which is ~11 times less than that of WTgp4, thereby revealing the importance of ATPase activity of helicase during DNA replication.

## 3. Discussion

The efficiency of replication progression depends on the coordination of the helicase and polymerase to perform duplex unwinding and nascent-strand DNA synthesis. The helicase is an essential component for most replisomes and has been proposed to be the driving motor in the unwinding of duplex parental DNA. In addition, many studies indicate that the polymerase can perform strand-displacement synthesis and play a vital role in separating base pairs at the fork [27]. However, how each motor contributes to the coupled unwinding remains elusive. Notably, our previous model of the T7 replisome suggested that the T7 helicase does not bind to the fork opening or the parental DNA [24]. Instead, the leading-strand polymerase encompasses a β-hairpin loop at the fork opening and a positively charged cleft with three lysine residues holding the parental duplex. As a result, the conformation of the parental DNA bound to gp5 is fixed, while the helicase appears to be flexible relative to gp5 and parental DNA. Here, our biochemical data confirmed the essential role of the β-hairpin loop and positively charged cleft on gp5. Deleting the loop or charge-reversal mutations in the positively charged patch inhibited gp5 strand-displacement synthesis, replisome assembly, and processive DNA replication, thereby highlighting the role of gp5 in unwinding downstream DNA.

Gp5 uses its β-hairpin loop to stack at the fork opening when the helicase and polymerase stay together to unwind the parental duplex. The location of the loop is reminiscent of the separation pin in many monomeric helicases [39,40]. This structural arrangement implies that the loop is essential for splitting the dsDNA. As shown in our results, deleting the β-loop reduces gp5 DNA binding, strand-displacement synthesis, gp4 loading and local unwinding, and processive DNA synthesis, which is consistent with its role in strand separation. Moreover, the β-loop is located between gp4 and gp5 in the T7 replisome. Although no direct interaction between the loop and gp4 has been observed, LDgp5 does eliminate the synergistic binding of gp4 to the fork substrate, which is similar to the ΔC mutant of gp4 [9]. LD gp5 has a similar affinity to fork DNA with various sizes of gaps, indicating that LD gp5 binding may not involve local parental DNA unwinding. Without the loop, gp5 may not be appropriately positioned at the fork to interact C-tail of gp4. On the other hand, we cannot exclude the possibility that the loop could still be involved in direct interaction with gp4, as the overall resolution of the replisome structure is low and several loops in gp4 are disordered. The β-hairpin loop on gp5 may have multiple functions. For instance, a recent study suggests that the β-hairpin loop is important for coordinating polymerase and exonuclease activity [34]. We therefore speculate that the β-hairpin loop might help hold the DNA template in place during exonuclease proofreading.

During DNA replication, the interactions between parental DNA and the DNA polymerase may help stabilize the replisome [41]. The structure of the gp5-DNA complex suggests that its three lysine residues form a positive patch and interact with the backbone of the parental DNA [24]. The K3D mutation in this study reduced its DNA binding ~3-fold, confirming its role in DNA interaction. Further, the K3D mutation had only a minor effect on DNA synthesis on a linear substrate but significantly reduced strand-displacement synthesis. Although the K3D gp5 could still promote gp4 binding, the DNA replication was significantly attenuated without the patch. This interaction may help orient the parental DNA for synergistic separation by gp4 and gp5. A similar positively charged cleft can be mapped in B-family T4/RB69 and Φ29 polymerases [28]. Moreover, with additional domains (TPR2) encircling the single-stranded DNA on the leading strand, Φ29 polymerase can catalyze efficient strand-displacement synthesis without a helicase [42,43,44]. Finally, a similar positively charged patch can be found in many monomeric helicases, where they help hold the dsDNA in place to allow proper unwinding [39].

Previous schematic illustrations of DNA replication always place the helicase at the replication fork, considering its motor activity. In eukaryotic helicases, the MCM NTD directly interacts with the parental DNA and provides a loop structure to stack at the fork opening [45]. However, the parental DNA is on the C-terminal side of bacterial and bacteriophage helicases, and no structural elements were found to interact with the parental DNA [24]. Various experiments have suggested that SF4 helicases are passive motors and work inefficiently while unwinding DNA [46,47,48]. Only in the presence of a polymerase can helicase unwinding be active and processive [7,44]. Although the gp4-gp5 interaction can help gp4 loading, deleting it only causes a moderate reduction of DNA synthesis [9]. In addition, switching of polymerases from different species still supports processive DNA synthesis [7]. Therefore, it is likely that the helicase only plays an accessory role in parental DNA unwinding, where it holds the unwound DNA in place and prevents backtracking of the replisome [7,27,44]. Interestingly, a recent single-molecule assay suggested that ATP hydrolysis by a bacterial helicase is not needed for replisome progression [38]. In the T7 system, a lower concentration of dTTP reduces the speed of the replisome [49,50]. Moreover, our RCA with EQgp4 indicated that dTTPase-coupled translocation is strictly required for efficient gp5 leading-strand DNA synthesis.

Our new biochemical data and extensive literature have enabled us to build a model for coupled helicase-polymerase action in bacterial and bacteriophage DNA replication. The polymerase plays a leading role in unwinding parental DNA. Incoming nucleotide-binding may provide the driving force for polymerase translocation along ssDNA [51,52]. To facilitate the steric displacement of the complementary strand, the polymerase uses a β-hairpin loop that stacks at the fork opening to promote base-pair separation. At the same time, the polymerase encompasses a positively charged patch that can hold and orient the parental DNA in place for duplex separation. These structural elements are reminiscent of those in a monomeric helicase, which can unwind dsDNA efficiently via ATP hydrolysis [39]. Although Φ29 polymerase can perform efficient strand-displacement synthesis alone due to its ring-like structure [43], most bacterial or phage polymerases have low-efficiency during strand-displacement synthesis, are sensitive to the base-pairing energy of the duplex, and exhibit reduced efficiency due to backtracking [27]. SSB can bind to the displaced strand and stimulate polymerase activities, but only to a limited extent [33]. The helicase, which binds to, and actively translocates along, the ssDNA promotes polymerase unwinding and makes it efficient and processive [8,9]. The direct interaction between the helicase and polymerase helps hold the two together to enable processive DNA synthesis, but may not be required for their coupling [7]. On the other hand, the polymerase is sensitive to various kinds of DNA damage and cellular stress [53]. If a polymerase stalls, the helicase will slow down, and the accumulated leading-strand ssDNA sends out signals for replication stress response and DNA repair [12].

## 4. Materials and Methods

### 4.1. Clone, Expression and Purification of gp5-trx Complex

The trxA, gp5, and gp4 were constructed and expressed similarly as in [24]. If brief, the DNA of the trxA and T7 gp5 (D5A, E7A) were cloned into PRSFDuet and PET15b vectors, respectively. The overlapping PCR constructed the plasmids WAgp5, LDgp5, and K3Dgp5 with designed primers (Integrated DNA Technologies^®^, Coralville, IA, USA), and the site mutagenesis was based on the plasmid-encoding WTgp5. All the constructs were verified by sequencing the whole protein-coding region. The thioredoxin (trxA) together with T7 gp5 (D5A, E7A) and its variants plasmid were co-transformed into E. coli BL21 (DE3) (Novagen). Bacteria were grown at 37 °C in 1 L Luria Broth until the value of OD 600 was 0.6–0.8. Isopropyl ß-D-1-thiogalactopyranoside (0.75 mM) was later added in the medium to induce protein expression. The expression was incubated at 16 °C and kept shaken for 18 h. The cells were collected and spun down at 6000 rpm for 40 min. Cell pellets of the trxA-T7gp5 complex and its variants were collected and mixed with lysis buffer containing 25 mM Tris-HCl, pH 7.5, and 50 mM KCl, 0.1 mM Ethylenediaminetetraacetic acid (EDTA), and 3 mM dithiothreitol (DTT). The collected mixture was later treated with sonication to break the cells. The sonicated pellets were spun down at high speed (19,000 rpm) for collection of the soluble fraction. Next, the supernatant was collected and filtered for loading onto the Heparin HP and MonoQ 10/100 GL column (both are with GE Healthcare) for purification. A buffer containing a low salt concentration including 25 mM Tris-HCl, pH7.5, 50 mM KCl, 0.1 mM EDTA, and 3 mM DTT was used for the equilibrium of the column and binding the protein to the beads of the columns. The protein was collected under a high-salt buffer containing 25 mM Tris-HCl, pH7.5, 1 M KCl, 0.1 mM EDTA, and 3 mM DTT. The collected protein was mixed with glycerol (50%) for storage in a −80 °C fridge.

### 4.2. Clone, Expression, and Purification of gp4 Complex

The DNA-encoding gp4 and its variants were cloned into a modified PET28a vector with histidine tags and a cleavage site for the prescission protease. The plasmids WTgp4ΔC and EQgp4ΔC were constructed by site mutagenesis based on the plasmids encoding WTgp4 and EQgp4, respectively. The T7 gp4 and its plasmid variants were transformed into C3013I cells derived from T7 Express lysY/Iq Competent E. coli (Novagen). Similar to gp5-trx, bacteria containing the gp4 expression vector were grown in Luria Broth until the value of OD 600 was 0.6–0.8, and 0.75 mM isopropyl ß-D-1-thiogalactopyranoside was used for protein induction followed by 18 h induction at 16 °C. Cell pellets containing the T7 gp4 and its variants were mixed with the buffer containing 25 mM Tris-HCl, pH 7.5, 1 M NaCl, 25 mM imidazole, 10 mM imidazole, and 3 mM DTT. After sonication and ultra-centrifugation (19,000 rpm), the supernatants were loaded onto the Histrap column (GE Healthcare). The column was equilibrated by a buffer containing 25 mM Tris-HCl (pH 7.5) and 1 M NaCl, 25 mM imidazole, 10 mM imidazole, and 3 mM DTT. After the supernatant was loaded onto the Histrap column (GE Healthcare), the column was washed by the washing buffer containing 25 mM Tris-HCl (pH 7.5) and 1 M NaCl, 50 mM imidazole, 0.1 mM EDTA, and 3 mM DTT. The protein was eluted by the elution buffer containing 25 mM Tris-HCl (pH 7.5) and 1 M NaCl, 500 mM imidazole, 0.1 mM EDTA, and 3 mM DTT. The eluted protein was mixed with a prescission protease at a 25:1 ratio at 4 °C for 2 h to remove the histidine tag. The solution was later diluted by a low-salt buffer containing 25 mM Tris-HCl (pH 7.5), 50 mM KCl, 0.1 mM EDTA, and 3 mM DTT for loading onto the MonoQ column (GE Healthcare). The protein (gp4 and its variants) was later eluted in a high-salt buffer containing 25 mM Tris-HCl (pH 7.5), 1 M KCl, 0.1 mM EDTA, and 3 mM DTT. The collected protein was mixed with glycerol (50%) for storage in a −80 °C fridge.

### 4.3. DNA Polymerase Assays

The DNA substrates for polymerases assays are listed in Table S1. The DNA substrates were annealed by incubating at 95 °C for 3 min and gradually cooling to 20 °C (1 °C drop per min) in the buffer, including 10 mM Tris (pH 7.5) and 50 mM NaCl. The DNA polymerase elongation reaction was performed by making mixture A, which contained 50 mM Tris (pH 7.5), 100 mM KCl, 0.1 mg/mL, 3 mM DTT, 12.5 nM of primer-template, 10 nM of gp5-trx and its variants; and mixture B containing 0.5 mM each of dATP, dCTP, dGTP, dTTP, 2.5 mM MgCl_2_ in advance. By mixing mixtures A and B, the reaction was initiated and was stopped by a quenching buffer (80% Formamide, 20 mM EDTA, 0.01% Bromophenol Blue) over time (0, 5, 15, 30, 60, 120, 240, 360 s) to estimate the dNTP-incorporation rate. The reaction mixture was later incubated in a 95 °C water bath for 5 min and cooled down on ice for 2 min. Samples were loaded into a 15% Urea-polyacrylamide gel for electrophoresis (250 V, 40 mA, 90 min) in the TBE buffer (100 mM Tris, 100 mM Boric acid, 2 mM EDTA). The electrophoresis results were analyzed and quantified by a scanner (Sapphire Biomolecular imager, Azure Biosystem^®^, Dublin, CA, USA) and Azure Spot (Azure Biosystem^®^).

### 4.4. Electrophoretic Mobility Shift Assays

The linear and fork templates (2 gaps) were prepared as in the DNA polymerase assay (Table S1). A 10 nM DNA substrate (0–2 gaps) (Table S1) and an increasing amount (5–600 nM) of gp5 variants (WTgp5, WAgp5, LDgp5, and K3Dgp5) were incubated for 30 min at 25 °C under a buffer containing 20 mM Hepes (pH 7.5 at 20 °C), 1 mM EDTA, 100 mM KCl, 0.1 mM βME, 5% *v*/*v* glycerol, 0.10 mg/mL BSA. For electrophoresis, reaction samples (20 μL) were loaded into 6% polyacrylamide gel at 150 V for 70 min. To perform the supershift test with EQgp4 in the mobility shift assay, 10 nM DNA substrate (2 gaps) (Table S1) and gp5 variants (60–100 nM) were incubated in advance at 25 °C for 15 min. Then an increasing amount of hexameric EQgp4 was added to each premixed aliquot (DNA-gp5 complex) for incubation at 25 °C for 15 min under the buffer containing 20 mM Hepes (pH 7.5 at 20 °C), 1 mM EDTA, 100 mM KCl, 0.1 mM βME, 5% *v*/*v* glycerol, 0.10 mg/mL BSA. Samples are loaded into wells of the 4% polyacrylamide gel for electrophoresis at 150 V for 70 min.

### 4.5. 2-Aminopurine (2-AP) Assay

The fluorescent intensity detected from the base-pair unwinding was through the fluorometer (Cary Eclipse Fluorescence Spectrophotometer, Agilent^®^, Santa Clara, CA, USA). The sample was excited at 315 nm (Slit Width: 10 nm), and the emission was measured at the interval between 340 and 400 nm (Slit Width: 10 nm). The fluorescent intensity is collected and integrated under a 340–400 nm wavelength, and the integrated results were present as columns in the statistics. Later, 200 nM DNA templates (See Table S1) and 200 nM proteins (EQgp4 variants, gp5 variants, and the mixture of Eqgp4 variants-gp5) variants, respectively, were mixed in a 100 μL cuvette under a reaction buffer containing 50 mM Tris (pH7.5), 100 mM KCl, 0.1 mg/mL, 3 mM DTT, 200 mM dTTP at 25 °C.

### 4.6. Rolling Circle Amplification

To form a single-stranded circle, a 70 nt oligo (Table S1) was phosphorylated with T4 polynucleotide kinase (37 °C) and then annealed via a splint oligo to later ligate its 5′ and 3′ end by T4 DNA ligase (16 °C). A splint oligo has the complementary sequence at the 5′ and 3′ end of the linearized 70 nt oligo. The 70 nt single-stranded DNA was purified with 10% urea polyacrylamide gel and then dried by a freeze dryer. Pellets collected during the drying process were later dissolved by water and desalted by the desalting column. The circular DNA was later annealed using a primer sequence with an overhang sequence of 40 nt at the 5′ end and 70 complementary nt. The ratio of primer to template was 1.5:1 in the annealing process. The DNA substrate was purified from 8% polyacrylamide gel (Table S1). The DNA synthesis reaction was initiated by mixing the minicircle (35 nM) with gp4 and gp5 variants, gp2.5 with 1 mM each of dATP, dCTP, dGTP, and dTTP in the buffer containing 40 μM Tris (pH 7.5), 2.5 mM MgCl_2_, 10 mM DTT, 0.1 mg/mL BSA, and 50 mM potassium chloride. [α-^32^P] dATP was used at 600 mCi/mmol as the indication to measure leading-strand synthesis. The gp4 and gp5 variants were premixed and incubated on ice for 5 min before being mixed into the reaction to make the final concentration of 250 nM. In the reaction mixture, gp2.5 was added to the final concentration of 4 μM. After the reaction was incubated in a water bath at 30 °C over time, the addition of 300 mM EDTA quenched the reaction. Samples were loaded into the alkaline agarose gel for electrophoresis (20 V, 8 mA, 24 h). A gel dryer (Model 583 and HydroTech™ Pump Gel Drying Complete Systems, BioRad^®^, Hercules, CA, USA) later dried the agarose gel for 2 h at 0.95 bar, 80 °C. Finally, we used the phosphoryl film for autoradiography, and the radioactivity was analyzed and quantified through the scanner.

## Figures and Tables

**Table 1:** K_d_ of WTgp5, WAgp5, LDgp5, and K3Dgp5, and DNA substrates with various gaps at the fork.

	WTgp5	WAgp5	LDgp5	K3Dgp5
Linear 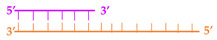	53 ± 5 nM	48 ± 3 nM	99 ± 3 nM	170 ± 6 nM
2gap 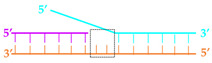	36 ± 5 nM	66 ± 4 nM	127 ± 11 nM	112 ± 18 nM
1gap 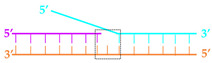	63 ± 4 nM	84 ± 16 nM	139 ± 23 nM	163 ± 2 nM
0gap 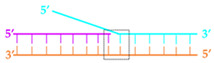	110 ± 13 nM	81 ± 5 nM	136 ± 9 nM	214 ± 9 nM

**Table 2:** K_d_ of the DNA-gp5 variants complexed with EQgp4 and EQgp4ΔC.

	DNA	DNA-WTgp5	DNA-LDgp5	DNA-K3Dgp5
EQgp4	24 ± 3 nM	8 ± 1 nM	25 ± 5 nM	6 ± 1 nM
Control EQgp4ΔC	30 ± 1 nM	26 ± 1 nM	/	/

## Data Availability

All materials and original data will be available upon request.

## Supplementary Materials

The following supporting information can be downloaded at: https://www.mdpi.com/article/10.3390/ijms23031342/s1.
