# Supplementary material for: DNA Polymerase-Parental DNA Interaction Is Essential for Helicase-Polymerase Coupling during Bacteriophage T7 DNA Replication
Source: Int J Mol Sci. 2022 Jan 25;23(3):1342. doi: 10.3390/ijms23031342 (PMC8835902; doi:10.3390/ijms23031342)
Supplement: Supplementary file 1 [file ijms-23-01342-s001.zip › ijms-1560738-supplementary.pdf]

International Journal of
Molecular Sciences

MDPI

Supplementary Materials:

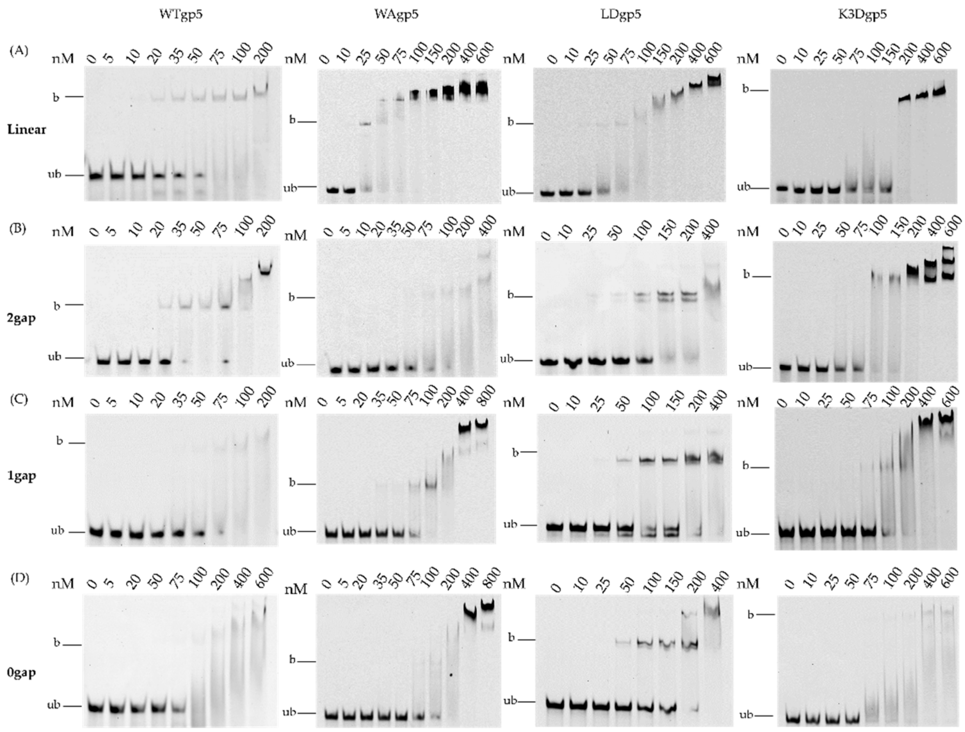

Figure S1. Electrophoretic mobility shift assay of gp5 variants and DNA templates. **(A)** EMSA gels showing binding affinity of gp5 variants (WT, WA, LD, K3D) with fixed concentration of linear DNA template (10 nM) and the increasing concentrations of gp5 variants. **(B-D)** EMSA gels showing binding affinity of gp5 variants (WT, WA, LD, K3D) with changing the gap sizes (2gap, 1 gap,0 gap) between the primer end and the fork on the DNA template. b, protein-bound DNA; ub, unbound DNA.

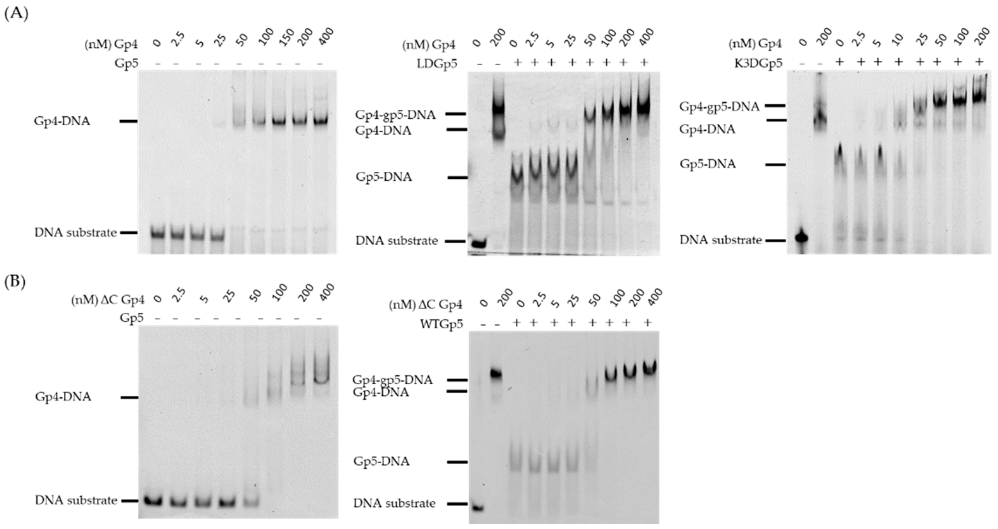

Figure S2. Super shift assay of the complexes of gp4-gp5-DNA. **(A)** EMSA result showing the binding affinity of EQgp4, EQgp4-LDgp5, and EQgp4-K3Dgp5 to fork

DNA template (2gap). **(B)** EMSA result showing the binding affinity of EQgp4ΔC, and EQgp4ΔC-WTgp5 to fork DNA template (2gap).

Table S1. DNA substrates for DNA polymerase assay,mobility shift assay, 2-AP assay ,and rolling circle assay.

**DNA substrates**	**Annealed Oligos**
Linear template-58nt 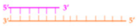	Cy5 26nt Primer+Temp58nt
Linear template-28nt 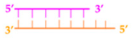	Cy5 26nt Primer+Temp28nt
Fork template 2gap 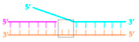	Cy5 26nt Primer+Temp58nt+Lag2gap
Fork template 1gap 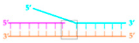	Cy5 26nt Primer+Temp58nt+Lag1gap
Fork template 0gap 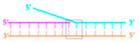	Cy5 26nt Primer+Temp58nt+Lag0gap
Oligos	Sequence
Cy5 26nt Primer	5′-/5cy5/GGATTATTTACATTGGCAGATTCACC-3′
Temp58nt	5′-TTATTAGTTATGATATTCATGTATAGTTATA TGGTGAATCTGCCAATGTAAATAATCC-3′
Temp28nt	5′-ATGGTGAATCTGCCAATGTAAATAATCC 3′
Lag2gap	5′TATTCATGTATAGTTATGTAGAGTTCCGAG ATCCGCGCTGCCAGTCGGCAGAGCC 3′
Lag1gap	5′TATTCATGTATAGTTATGTAGAGTCCG AGATCCGCGCTGCCAGTCGGCAGAGCC 3′
Lag0gap	5′TATTCATGTATAGTTATGTAGAGACCGAGA TCCGCGCTGCCAGTCGGCAGAGCC 3′
DNA substrates	Oligos annealed
+1 lag no gap 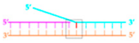	2-AP lag+(no gap first bp Primer)+(no gap first bp Lead)
+2 lag no gap 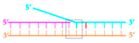	2-AP lag+(no gap second bp Primer)+(no gap second bp Lead)

+1 lag 1gap 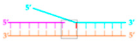	2-AP lag+(1 gap first bp Primer)+(1 gap first bp Lead)
+2 lag 1gap 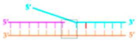	2-AP lag+(1 gap second bp Primer)+(1 gap first bp Lead)
+1 lag 2gap 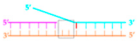	2-AP lag+(2 gap first bp Primer)+(2 gap second bp Lead)
+2 lag 2gap 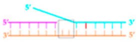	2-AP lag+(2 gap second bp Primer)+(+2 Lead 2 gap)
2-AP lag	5′TTTTTTTTTTTTTTTTTTTTTTTTTTTTTG/i2AMP/TC GCTAGTGCTGTGATCGATTGCTTGTA 3′
No gap first bp Primer	5′ CTAGTAACAGTGTGAACTTGACGACATAGCGA 3′
No gap second bp Primer	5′ CTAGTAACAGTGTGAACTTGACGACATAGCGTA 3′
1 gap first bp Primer	5′ CTAGTAACAGTGTGAACTTGACGACATAGCG 3′
1 gap second bp Primer	5′ CTAGTAACAGTGTGAACTTGACGACATAGCGT 3′
2 gap first bp Primer	5′ CTAGTAACAGTGTGAACTTGACGACATAGCG 3′
2gap second bp Primer	5′ CTAGTAACAGTGTGAACTTGACGACATAGCG 3′
No gap first bp Lead	3′GATCATTGTCACACTTGAACTGCTGTATCGCTTAGC GATCACGACACTAGCTAACGAACAT 5′
No gap second bp Lead	3′GATCATTGTCACACTTGAACTGCTGTATCGCAT CTAGCGATCACGACACTAGCTAACGAACAT 5′
1 gap first bp Lead	3′GATCATTGTCACACTTGAACTGCTGTATCGCTTA GCGATCACGACACTAGCTAACGAACAT 5′
1 gap second bp Lead	3′GATCATTGTCACACTTGAACTGCTGTATCGCATCTA GCGATCACGACACTAGCTAACGAACAT 5′
2 gap first bp Lead	3′GATCATTGTCACACTTGAACTGCTGTATCGCTGTA GCGATCACGACACTAGCTAACGAACAT 5′
2gap second bp Lead	3′GATCATTGTCACACTTGAACTGCTGTATCGCTGCTA GCGATCACGACACTAGCTAACGAACAT 5′
Minicircular DNA with Primer 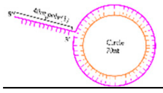	Circular MiniC1+MiniC2 Primer

MiniC1	5′ GACCATATCCTCCACCCTCCCCAATATTGACCATCAA CCCTTCACCTCACTTCACTCCACTATACCACTC 3′
MiniC Splint	5′ GGATATGGTCGAGTGGTATA 3′
MiniC2 Primer	5′ $(T)_{40}$GGTGGAGGATATGGTCGAGTGGTATAGTGGAGTG AAGTGAGGTGAAGGGTTGATGGTCAATATTGGGGAG 3′
